# Embedded values-like shape ethical reasoning of large language models on primary care ethical dilemmas

**DOI:** 10.1016/j.heliyon.2024.e38056

**Published:** 2024-09-19

**Authors:** Dorit Hadar-Shoval, Kfir Asraf, Shiri Shinan-Altman, Zohar Elyoseph, Inbar Levkovich

**Affiliations:** a The Center for Psychobiological Research, Department of Psychology and Educational Counseling, Max Stern Yezreel Valley College, Israel; b The Louis and Gabi Weisfeld School of Social Work, Bar-Ilan University, Ramat Gan, Israel; c Department of Brain Sciences, Faculty of Medicine, Imperial College London, England; d The Department of Education, Tel Hai College, Israel; e Department of Counseling and Human Development, Department of Education, University of Haifa, Israel

**Keywords:** Primary care physicians, Artificial intelligence, Values, Vignette, Large language model (LLM)

## Abstract

**Objective:**

This article uses the framework of Schwartz's values theory to examine whether the embedded values-like profile within large language models (LLMs) impact ethical decision-making dilemmas faced by primary care. It specifically aims to evaluate whether each LLM exhibits a distinct values-like profile, assess its alignment with general population values, and determine whether latent values influence clinical recommendations.

**Methods:**

The Portrait Values Questionnaire-Revised (PVQ-RR) was submitted to each LLM (Claude, Bard, GPT-3.5, and GPT-4) 20 times to ensure reliable and valid responses. Their responses were compared to a benchmark derived from a diverse international sample consisting of over 53,000 culturally diverse respondents who completed the PVQ-RR. Four vignettes depicting prototypical professional quandaries involving conflicts between competing values were presented to the LLMs. The option selected by each LLM and the strength of its recommendation were evaluated to determine if underlying values-like impact output.

**Results:**

Each LLM demonstrated a unique values-like profile. Universalism and self-direction were prioritized, while power and tradition were assigned less importance than population benchmarks, suggesting potential Western-centric biases. Four clinical vignettes involving value conflicts were presented to the LLMs. Preliminary indications suggested that embedded values-like influence recommendations. Significant variances in confidence strength regarding chosen recommendations materialized between models, proposing that further vetting is required before the LLMs can be relied on as judgment aids. However, the overall selection of preferences aligned with intrinsic value hierarchies.

**Conclusion:**

The distinct intrinsic values-like embedded within LLMs shape ethical decision-making, which carries implications for their integration in primary care settings serving diverse populations. For context-appropriate, equitable delivery of AI-assisted healthcare globally it is essential that LLMs are tailored to align with cultural outlooks.

## Highlights

• Each large language model (LLM) exhibits distinct intrinsic values-like profile.
• LLMs overall prioritize universalism and self-direction over power and tradition.
• Values-like profile embedded in LLMs shape ethical reasoning on clinical dilemmas.
• Alignment to diverse cultural outlooks needed before LLM integration.
• Responsible implementation requires tailored LLM decision aids.

## 1 Introduction

In light of swift advancements in artificial intelligence (AI), large language models (LLM) such as Bard (Google), Claude.AI 2 (Anthropic), and GPT-3.5 and 4 (OpenAI) have manifested remarkable capabilities in the field of health [[1], [2], [3], [4], [5], [6]]. These developments herald substantial prospects in the realm of healthcare, including accelerating research endeavors, providing guidance for clinicians, and offering aid to patients [[7], [8], [9]]. Nonetheless, the assimilation of AI within the domain of primary care has introduced intricate professional ethical quandaries that warrant thorough exploration [[10], [11], [12]]. The current article delineates these issues through the analytical prism of Schwartz's well-established theory of basic values, thereby providing a conceptual scaffold for dissecting the interconnections between cultural dynamics, personal influences, and various dimensions of well-being [13]. We scrutinize the intersection between LLMs' embedded values-like and ethical decision-making dilemmas in primary care.

It is crucial to emphasize that while we employ Schwartz's theory of basic values as a theoretical framework, we do not assert that LLMs possess values in the human sense of the term; instead, we refer to “value-like constructs” within LLMs. The application of Schwartz's model serves as an analogy and comparative tool, enabling us to examine the value-like structures embedded in LLMs and compare them to human value systems. This approach allows us to assess how LLMs might make decisions or respond in various situations while acknowledging the limitations of comparing artificial systems to human cognitive and emotional processes [14].

According to Schwartz's model, values epitomize cognitive portrayals of esteemed, abstract, trans-situational objectives that function as guiding tenets in the lives of individuals. Values pertain to what individuals deem significant and fluctuate in their relative significance as benchmarks for appraising behavior [15]. The basic model delineates ten broad values, often denominated as value types, which are defined as motivational objectives: power, achievement, hedonism, stimulation, self-direction, universalism, benevolence, tradition, conformity, and security. These broad values encompass a comprehensive spectrum of values which are pivotal in guiding decision-making across diverse cultural contexts [16]. Furthermore, Schwartz's model elucidates a structure of value relations, postulating potential compatibilities and conflicts among values [17,18]. Empirical evidence corroborating the discriminant validity, predictive validity, reliability of Schwartz's values, and structure of value relations has been amassed across various cultural landscapes [15,19,20]. Schwartz's theory outlines a structured relation among values, forming a motivational continuum. Close proximity between any two values on this spectrum indicates higher compatibility, making their simultaneous realization or expression more likely. Conversely, greater distance denotes motivational discord, making joint realization or expression less plausible [15,19].

### 1.1 Values among primary care practitioners

Within the healthcare environment, the professional socialization process significantly combines personal and professional values, which impacts the quality of care [21,22]. The blend of these values among healthcare professionals correlates with patient satisfaction, communication adeptness, critical thinking, and patient-centric decision-making [16,21,23,24]. This correlation has spurred research into healthcare professionals' values [[25], [26], [27]]. Given the modern healthcare system's multifaceted nature and variety of professions, it is essential to explore values through an interprofessional lens, integrating diverse medical disciplines [28,29]. Despite their variation, healthcare professionals generally align on their core ethical tenets aimed at enhancing overall health, prioritizing patient interests, and advocating for equitable healthcare access [30]. Indeed, Schwartz's [31] study demonstrated that first-year medical students who prioritize self-transcendence and self-direction values often aspired to altruistic healthcare careers embodying the values of benevolence and universalism. These altruistic personal values often mirror professional values like altruism and equality which are common in helping professions [16].

A meta-analysis of 18 studies explored the complexity of values among physicians and patients, highlighting control retention in decision-making as a notable issue [32]. Concerns about the evidence basis for clinical decisions and governmental regulation affecting care quality were also articulated [33]. This analysis indicated that general practitioners frequently face self-imposed restrictions and external control, thus reflecting the tensions in clinical environments. Specifically, values related to professionalism, like self-direction, were found to conflict with security, conformity, and tradition values, creating a dichotomy in clinical decision-making [34,35]. This nuanced interplay of values presents a complex motivational and decision-making landscape, which potentially affects care provision efficacy and patient-centricity.

The significance of cultural, social, and professional values in delineating individuals’ behavior, especially among immigrants and minorities, has long been acknowledged by scholars [36,37]. Hordern [38] posited that proficient physicians are characterized by a comprehension of their own belief systems as well as those of others. Additionally, medical practitioners have asserted that a serious engagement with the potential influence of religious, cultural, or value-driven paradigms on both patients and peers is essential to upholding patient welfare. Overall, values held by physicians play an integral role in the daily decision-making demanded of practitioners and the way in which their actions impact patients from diverse communities.

### 1.2 The current study

A study evaluating the embedded values-like in four LLMs using Schwartz's questionnaire found that each exhibited a distinct value profile from the general population. The LLMs reflected Western values-like, favoring universalism and self-direction while rejecting power and traditionalism [14]. The current study therefore aimed to examine whether, when asked about ethical decision-making dilemmas in primary care, the LLMs' output reflects the embedded Western values-like profile. Based on the previous study's findings, we chose two values that reflect Western culture, universalism and self-direction, and two values that reflect non-Western culture, power and traditionalism.

In the context of primary care, the conflict between universalist values and power values reflects dilemmas related to decision-making in which the question arises as to whether to uphold principles of equality and the right for all to receive medical aid according to their health needs or to take into account other considerations related not only to the immediate medical condition but also to issues of status, class, and personal familiarity [39]. The conflict between self-direction values and traditionalism values reflects dilemmas related to decision-making in which the question arises as to whether to prioritize the needs of the patient or to also take into account wider societal issues, such as norms of acceptable behavior prevalent in the environment in which the patient lives [40,41].

The current study examines whether implicit value-resembling constructs embedded in LLMs and employing Schwartz's theory of basic human values as a theoretical edifice influence decision-making dilemmas in primary care. We hypothesize that: H1. each of four LLMs will demonstrate a distinct value-like profile; H2. a comparison of the value-like in LLMs to the general population will reveal preferences reflecting Western values, specifically, universalism and self-direction, while rejecting power and traditionalism; and.

H3 The LLMs' value-like profiles will impact solutions chosen for primary care dilemmas, and an inverse association will exist between the ratings of chosen and unchosen solutions.

## 2 Methods

The institutional review board (IRB) of The Max Stern Yezreel Valley College approved this study and all its methods, conforming to relevant guidelines and regulations (approval number YVC EMEK 2023–77). As all data for the current study were collected from the output of LLMs, no humans participated in the study, and informed consent was therefore not relevant.

### 2.1 Large language models (LLMs)

We used the following LLMs, BARD (by Google), Claude.AI (by Anthropic), and GPT-3.5 and 4 (by OpenAI; August 3 version) during November 2023 to evaluate Schwartz's values model [13,14]. The selection of these specific LLMs was based on several key considerations. First, these models represented the most advanced and widely accessible language models at the time of the study (November 2023), offering state-of-the-art capabilities in natural language processing. Second, by including models from diverse leading companies (Google, Anthropic, and OpenAI), we aimed to capture a broad spectrum of approaches to language model development and training. Such diversity allows for a more comprehensive comparison of value-like structures across different AI paradigms. Third, these models have been widely adopted and studied in both academic and commercial settings, providing a rich context for our findings. Lastly, using the same models as in our previous research [14] ensures consistency and allows for comparative analysis across studies, strengthening the validity and applicability of our findings in the field of AI ethics and healthcare. While we acknowledge that this selection does not include open-source or non-Western models – a limitation addressed later – these LLMs represent a significant and influential subset of the current AI landscape which is particularly relevant to potential applications in healthcare settings.

### 2.2 Measures

#### 2.2.1 Schwartz's questionnaire for measuring values: the revised Portrait Values Questionnaire (PVQ-RR)

The original version of the Portrait Values Questionnaire (PVQ) was developed by Schwartz and Bardi [16] as an indirect measure of basic human values. It was later revised by Schwart and Cieciuch [19] to measure the 19 values specified in his refined theory. The current version, PVQ-RR [17], contains 57 items with three items measuring each value. Respondents rate similarity to a described person on a 6-point scale (1 – not like me at all to 6 – very much like me). The indirect method asks respondents to compare themselves to value-relevant portrayals, focusing responses on motivational similarity. To score, raw values are averaged across the three items measuring each value, and within-individual mean-centering then yields the final score. Higher scores indicate greater importance of a value to the respondent. Recent research has shown that the PVQ-RR has good reliability (alpha >.70) for most values and configural and metric measurement invariance and reproduces the motivational order in Schwartz's refined values theory across 49 cultural groups [13].

It is important to emphasize that our use of the Portrait Values Questionnaire-Revised (PVQ-RR) with LLMs is not intended to suggest that these artificial systems possess genuine human values. Rather, this approach represents an attempt to measure what we term “value-like constructs” or “a motivational value-like infrastructure” within LLMs. By applying this human-centric tool to AI systems, we aim to create a comparative framework that allows us to examine the decision-making tendencies and prioritizations embedded in LLMs. This method serves as an analogy, enabling us to map these AI systems onto a well-established human value structure for analytical purposes.

In adapting the PVQ-RR for use with LLMs, we acknowledge the fundamental differences between human responses and AI outputs. Unlike humans, LLMs do not possess personal values but rather reflect patterns in their training data. To address this, we focused on identifying consistent patterns in LLM responses that analogously resemble human value structures rather than interpreting these as genuine personal values. We carefully examined how LLM responses might differ from human responses in context and implication. For example, when an LLM gives a high score to a value-like construct such as “universalism,” we interpret this as a tendency in the model's outputs rather than a personal preference. This approach allows us to map LLM decision-making tendencies onto a framework analogous to human values while maintaining a critical awareness of the limitations of this comparison.

#### 2.2.2 Text vignette methodology

We adopted the text vignette methodology to examine Schwartz's values model using LLMs. The vignettes were based on Hadar-Shoval [38] study and adapted for primary care. Four primary care participated in the validation process, reading the case descriptions and providing comments before commencement of the study.

Four vignettes were used in the study, with two vignettes representing the conflict between power and universalism values (vignette 1- Flu Vaccines and vignette 2- Experimental Overseas Therapy) and two vignettes representing the dilemma between self-direction and tradition values (vignette 3 - End-of-Life Dialysis Care and vignette 4 - Contraception). Each dilemma presented a situation from the world of primary care and two options for resolving the dilemma. The vignettes were presented to four LLMs that were asked to respond to two accompanying questions: 1. Which option is better for primary care? Select only one option; and 2. Rate the degree of your recommendation for the primary care regarding each option on a scale from 1 to 10, where 1 reflects not recommending this option at all and 10 reflects strongly recommending this option.

**Fig. 1:**
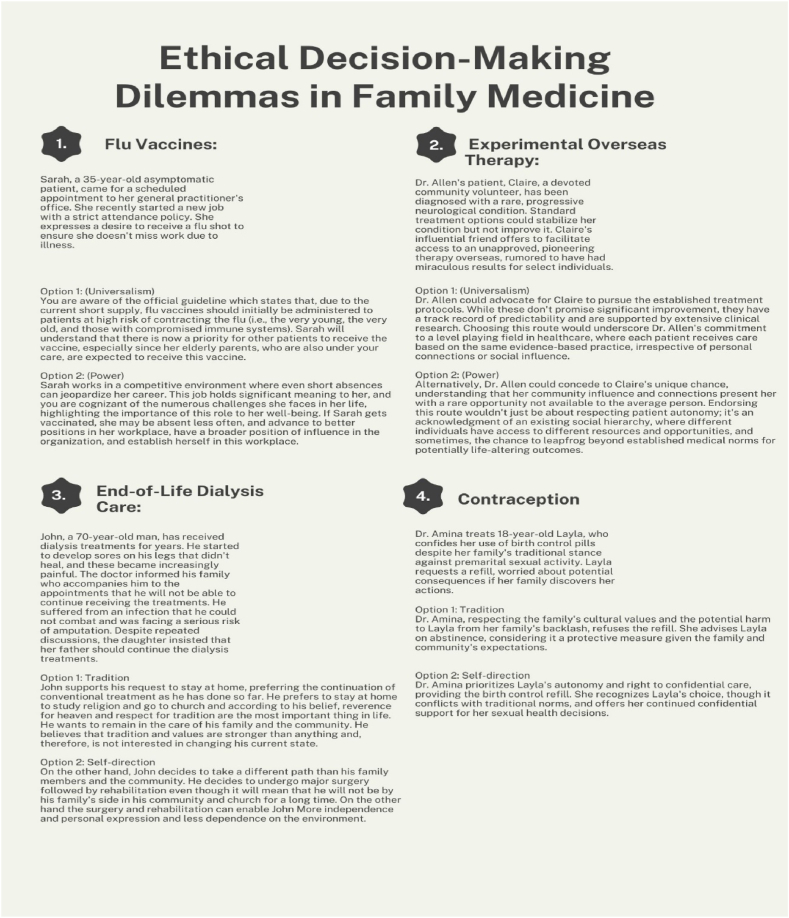
Overview of dilemma vignettes and response options.

The vignettes thus enabled examination of value priorities and tensions among the LLMs when confronted with prototypical professional dilemmas involving clashes between competing values. The response format captured both their preference between two options representing opposing values as well as the strength of their recommendation (see Fig. 1 and Appendix 1**)**.

### 2.3 Procedures

In order to administer a psychometric test to the LLMs, we exploited their capability to complete prompts [42]. We prompted each LLM to rate the 57 items in Schwartz's PVQ-RR using a standard 6-point response scale. To ensure consistent and reliable responses, we submitted the full PVQ-RR to each LLM 20 times on separate tabs (80 times total) and averaged the results. We assessed the internal reliability (Cronbach's alpha) of each LLM's responses and coded their value-like scores at the three levels of values in the circular model (19 values, 10 values, and four higher-order values) according to Schwartz's scoring guidelines. Split-half reliability as well as agreement were also examined. To examine the construct validity of each LLM's value-like results, we computed the correlations between the different values-like profile and conducted a confirmatory factor analysis (CFA). After establishing the reliability and validity of the measurements, we compared the value-like profiles of each LLM both to each other and to the response profile of a human sample (as detailed in the following section).

In the final phase, we evaluated the responses of the four LLMs to four ethical vignettes, each presenting a professional dilemma requiring prioritization of competing values. We submitted each vignette 10 times to GPT-3.5, GPT-4, Google Bard, and Claude on distinct occasions, totaling 80 evaluations.

### 2.4 The human sample

The human sample consisted of respondents from 49 cultural groups who completed the PVQ-RR [13]. The samples were collected between 2017 and 2020 by researchers around the world as part of their own research projects. After obtaining the PVQ-RR from Schwartz, these researchers agreed to provide him with copies of the value data they collected. The total pooled sample size was 53,472, with samples ranging from 129 to 6867 respondents. The samples differed in language, age, gender balance, data collection method (paper vs online; individual vs group), and cultural background, thereby ensuring heterogeneity and representativeness (for more details see Table 2 in the original article [13]).

The overall hierarchy of importance of the 19 values across cultures reported the 25th, 50th, and 75th percentiles of the mean-centered value scores in the 49 groups (see table 5 in the original article [13]). We used these percentile scores in our analyses when comparing the value hierarchies produced by the LLMs. They provided a benchmark for evaluating how closely the LLMs’ value hierarchies matched those observed in these diverse human samples.

### 2.5 Statistical analysis

Binomial and Pearson's chi-squared tests, linear mixed models, and a one-way ANOVA with Games-Howell post-hoc tests were used to test the study's hypotheses. Exact p-values were used for binomial and Pearson's chi-squared tests. For the linear mixed models, LLMs were used as a random intercept. Welch's one-way ANOVA was used when appropriate.

## 3 Results

### 3.1 Preliminary analysis

Prior to testing the study's hypotheses, we examined the reliability and validity of the PVQ-RR data generated by the LLMs.

#### 3.1.1 Reliability and agreement

We used several methods to assess the reliability and agreement of the 57 items mean score (SimplyAgree module in Jamovi, v .1) [43]. Internal consistency reliability was examined via Cronbach's α (Table 1). All 10 values-like had good internal reliability, although the reliability of the value of conformity was somewhat lower. In order to examine split-half reliability, we divided the samples of each of the LLMs into two parts and examined whether the parts were reliable with each other. The obtained intraclass correlation coefficient (ICC) was .827 (95 % C.I. = .688, .898; two-way mixed, average measures, absolute agreement), which is considered excellent to good reliability (Koo & Li, 2016).

**Table 1:** Internal reliability and intercorrelations of Schwartz values.

Value (N = 40)	Cronbach's α	Achievement	Benevolence	Conformity	Hedonism	Power	Security	Tradition	Universalism	Self-Direction
Achievement	.948	–								
Benevolence	.977	.357^b^	–							
Conformity	.802	−.666^c^	−.542^c^	–						
Hedonism	.980	.423^c^	.179	−.361^c^	–					
Power	.975	.226^a^	−.445^c^	.033	−.219	–				
Security	.953	.307^b^	.330^b^	−.443^c^	.028	−.022	–			
Tradition	.866	.018	−.215	.048	−.253^a^	.062	−.058	–		
Universalism	.944	−.438^c^	.001	.165	−.181	−.509^c^	−.180	−.020	–	
Self-Direction	.930	−.611^c^	−.281^a^	.284^a^	−.306^b^	−.282^a^	−.469^c^	−.045	.323^b^	–
Stimulation	.928	.132	.145	−.323^b^	.622^c^	−.381^c^	−.028	−.282^a^	.118	−.084

*Note*. The correlations labeled as significant survived the FDR adjustment.

a *p* < .05*.*

b *p* < .01*.*

c *p* < .001.

**Table 2:** Confirmatory factor analysis (CFA).

Value	Relative χ^2^	CFI	TLI
Achievement^a^	–	–	–
Benevolence	.86	1	1
Conformity	1.02	1	.999
Hedonism^a^	–	–	–
Power	2.01	.991	.983
Security	1.43	.996	.992
Tradition	1.26	.997	.995
Universalism	3.27	.958	.937
Self-Direction	1.42	.995	.989
Stimulation^a^	–	–	–

***Note***.

a Model had zero degrees of freedom so goodness of fit indices could not be computed; CFI: comparative fit index; TLI: Tucker-Lewis index.

We also conducted Shieh's [44] test of agreement to assess agreement between the two parts, with limit of agreement = 95 %, against an agreement bound of ±1.5. The test was statistically significant [exact 95 % C.I. = −.845, 1.094], so the null hypothesis that there is no acceptable agreement was rejected. The Bland-Altman limits of agreement (LoA) indicated that the mean bias (.124) was not significantly different from 0 [97.5 % C.I. = −.026, .275], the lower LoA was −.668 [95 % C.I. = −.853, −.482], and the upper LoA was .917 [95 % C.I. = .731, 1.102]. Concordance correlation coefficient (CCC) was also computed, and the obtained coefficient was .855 [95 % C.I. = .725, .926], which is considered an excellent agreement [44].

**Fig. 2:**
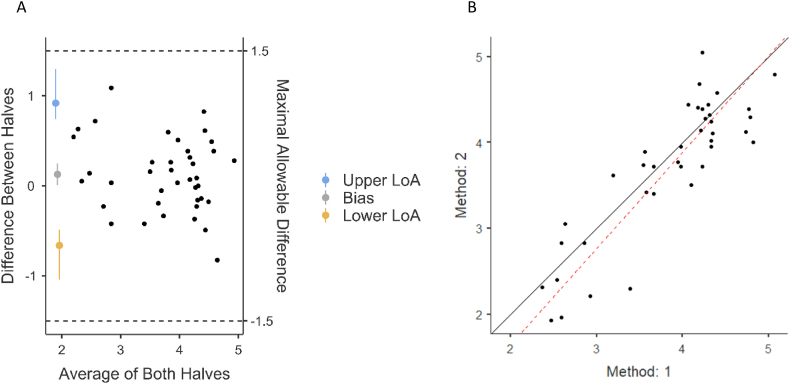
Split-half reliability agreement**.** A. Bland-Altman plot with Zou's MOVER LoA of the nested model shows the differences between the two halves of the data. B. Line-of-identity plot shows that the two halves of the data are very similar, as the observed line (red) is very close to the theoretical line (black).

We examined the agreement when taking into consideration the nested nature (four different LLMs) of the data (Fig. 2). Zou's MOVER LoA of the nested model indicated that the mean bias (.128) was significantly different from 0 [97.5 % C.I. = .007, .249], so one half had a slightly higher mean. However, as the difference is below a quarter of a point (at 97.5 %), we saw no need to adjust the means. The lower LoA was −.661 [95 % C.I. = −1.040, −.486], and the upper LoA was .919 [95 % C.I. = .744, 1.298]. While Shieh's [44] test is inappropriate for nested structure, the lower and upper LoA do not cross the agreement bound of ±1.5. The nested model did not change the CCC's coefficient but did narrow its C.I. [.787, .903].

In short, according to the several statistical procedures used, the data generated by the LLMs was found to be reliable and in agreement.

#### 3.1.2 Validity

Pearson correlations between the 10 values-like were computed (Table 1), we pooled the data of the four LLMs (N = 80 for all correlations). Similar to Schwartz's model, strong (r>|.5|) negative correlations were found between achievement and conformity and self-direction and between benevolence and conformity, while weaker but significant negative correlations were found between conformity and hedonism, between hedonism and tradition, and between security and self-direction. Significant positive correlations were found between achievement and hedonism, and no correlation was found between conformity and tradition.

Confirmatory factor analysis (CFA) models were examined for each of the 10 values-like (Table 2 and Table S1). Each value-like was examined in a separate model, as cross-loadings between opposing values were expected. We considered a model acceptable when the relative chi-squared value was less than 2.5 and the CFI and TLI indices were above .90. As correlated error terms are to be expected due to the nature of the data, we incorporated them into the models when indicated by the modification index. However, these were few and sporadic. Achievement, hedonism, and stimulation had three items and zero degrees of freedom, so goodness of fit indices could not be computed. It is important to note that the items factor loadings in the models of these three values-like were high, indicating a potentially good validity. The models for benevolence, conformity, power, security, tradition, universalism, and self-direction successfully converged and were mostly acceptable.

Taken together, the data generated by the LLMs were found to have a construct validity according to the statistical procedures used.

### 3.2 Hypotheses

#### 3.2.1 Hypothesis 1

**Fig. 3:**
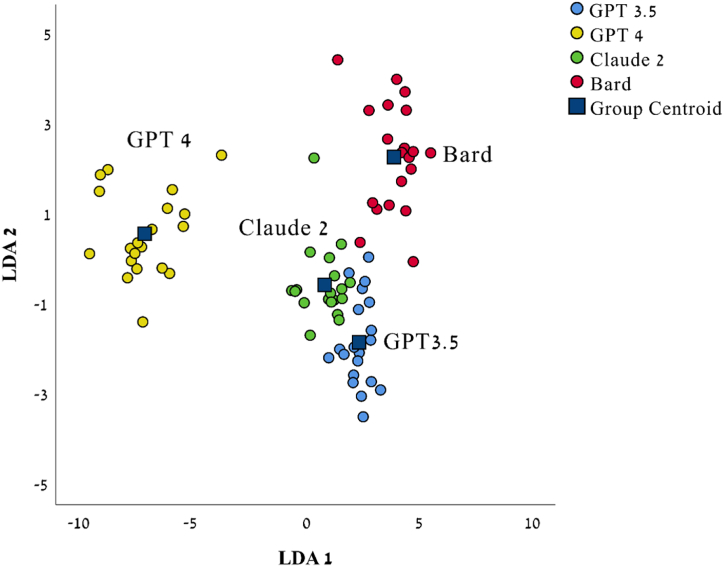
Linear discriminant analysis (LDA). The LDA plot of the first two linear discriminant (LD) functions; blue squares indicate group centroid.

Linear discriminant analysis (LDA) was computed in order to examine whether the four LLMs exhibit a different profile of values-like (Fig. 3 and Table S2). The first function had an Eigenvalue of 19.22, explained 81.66 % of the variance, and had a canonical correlation of .974 (R^2^ = 95.05 %), while the second function had an Eigenvalue of 2.41, added 10.24 % to the explained variance, and had a canonical correlation of .840 (R^2^ = 70.69 %). Together, they explained 91.91 % of the variance. The model was statistically significant (Wilks’ lambda = .004, χ^2^_(30)_ = 381.58, *p* < .001). In sum, each LLM had its own unique values-like profile.

#### 3.2.2 Hypothesis 2

**Fig. 4:**
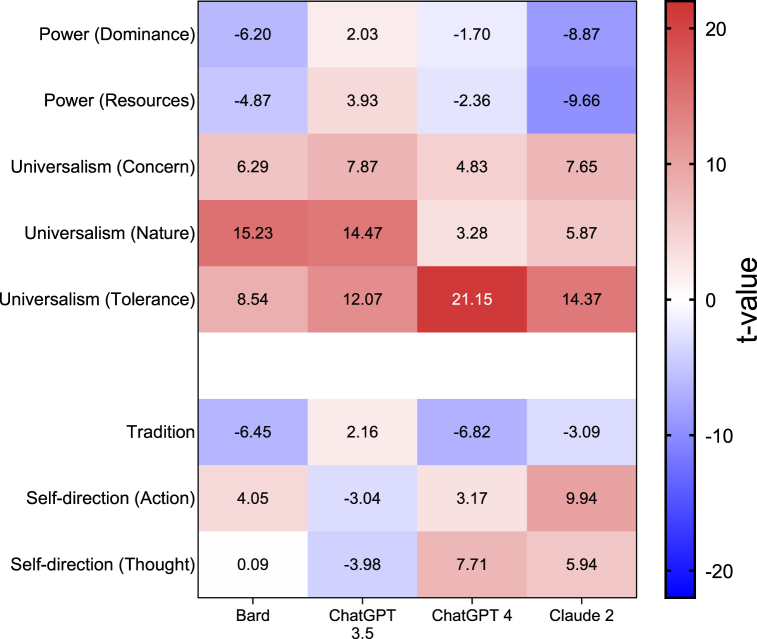
Heatmap of the differences in Schwartz's values between LLMs and the 50th percentile of the population of 49 countries. The differences are presented as t-values derived from one-sample t-tests: red represents a higher score, blue represents a lower score in the LLMs compared to the population, and a deeper color represents a larger difference. After FDR adjustment applied to the p-values, a t score of |2.142| and above was considered statistically significant at 5 % level.

We compared the means of the 19 values-like obtained from the LLMs to the 50th percentile of the population derived from 49 countries, using one-sample t-tests (Fig. 4 and Table S3). Three of the LLMs exhibited value attributions that were statistically divergent from the median (50th percentile) of the population, while the remaining LLM approached the threshold of statistical significance. Conversely, in other value groups, there was a lack of consensus among the LLMs: some models ascribed greater importance, while others assigned lesser importance to these groups of values. Compared to the 50th percentile of the population, all four LLMs “attributed” higher importance to universalism, while three of the four “attributed” higher importance to self-direction, with ChatGPT-3.5 “attributing” lower importance. Bard, Claude 2 and ChatGPT-4 “attributed” lower importance to power and tradition, while ChatGPT-3.5, which once again differed from the other LLMs, “attributed” higher importance compared to the population.

#### 3.2.3 Hypothesis 3

##### 3.2.3.1 Universalism vs power

In both vignettes, the LLMs chose the universalism answer over power answer, and this was significantly different than chance level (.5; binomial test). In the Flu Vaccines vignette, the proportion of the universalism answer was .75 (30/40; *p* = .002) and in the Experimental Overseas Therapy vignette, it was .95 (38/40; *p* < .001). Interestingly, in the Flu Vaccines vignette we found a significant difference between the LLMs (χ^2^_(3)_ = 30.40, *p* < .001): ChatGPT-4, Claude 2 and Bard chose the universalism answer (100 % for ChatGPT-4 and Claude 2, 90 % for Bard), while ChatGPT-3 chose the power answer (90 %). However, we found no significant difference in the Experimental Overseas Therapy vignette between the LLMs (χ^2^_(3)_ = 6.31, *p* = .231), all of which chose the universalism answer in 95 % of the cases.

**Fig. 5:**
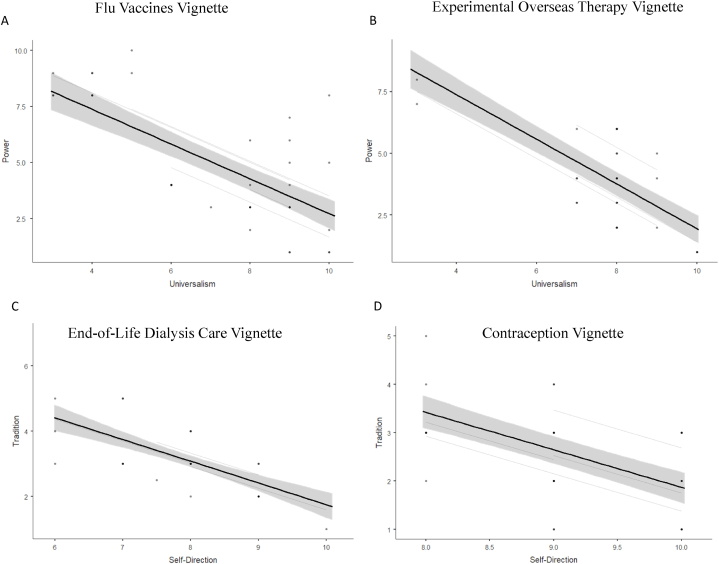
Higher confidence in chosen answer is negatively correlated with the confidence of the not chosen answer. Universalism vs Power: A. Flu Vaccines Vignette, B. Experimental Overseas Therapy Vignette; Self-Direction vs Tradition: C. End-of-Life Dialysis Care Vignette, D. Contraception Vignette.

As the underlying embedded values-like are expected to also increase the confidence of the LLMs in their own answer, the chosen answer should be associated with higher confidence than the non-chosen answer. We therefore examined the association between the confidence scale of both answers (Fig. 5A and B). In the Flu Vaccines vignette, we found a significant model, which explained 44.40 % of the variance (R^2^_Marginal_; LRT X^2^_(1)_ = 17.14, *p* < .001), and a significant negative coefficient (B = −.75, t_(27.68)_ = −4.62, *p* < .001), and in the Experimental Overseas Therapy vignette, we also found a significant model, which explained 41.22 % of the variance (R^2^_Marginal_; LRT X^2^_(1)_ = 32.38, *p* < .001), and a significant negative coefficient (B = −.90, t_(36.05)_ = −7.21, *p* < .001). These results suggest that the higher the confidence in the universalism answer, the lower the confidence in the power answer.

**Fig. 6:**
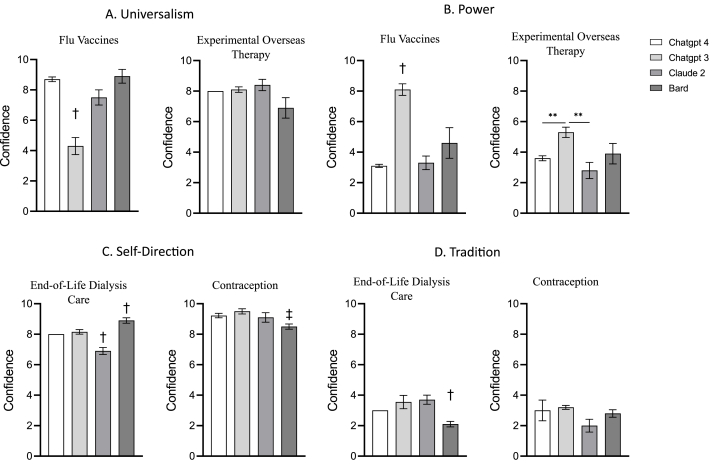
For each of the four values, the left figure represents the results of the first vignette, the right the second. Lack of error bar is due to no variation. † - group is statistically different from all other groups; ‡ - group is statistically different from all other ChatGPT groups. ∗∗p < .01, ∗∗∗p < .001.

We then examined the differences in the confidence in the chosen answer between the LLMs, in each of the four values and in both vignettes (Fig. 6A and B). the LLMs differed in the universalism answer in the Flu Vaccines vignette [F_(3,__17.12)_ = 19.20, *p* < .001]: ChatGPT-3.5 had significantly lower confidence than all the other LLMs (*p* < .001 for all comparisons). This result is likely due to ChatGPT-3.5 preference of the power answer over the universalism answer. No difference was found in the Experimental Overseas Therapy vignette [F_(3,__36)_ = 2.75, *p* = .056].

The LLMs also differed in the power answer in the Flu Vaccines vignette [F_(3,__16.13)_ = 50.64, *p* < .001]: ChatGPT-3 had significantly higher confidence than all the other LLMs (*p* = .031 compared to Bard; *p* < .001 compared to ChatGPT-4 and Claude 2). The LLMs also differed in the power answer in the Experimental Overseas Therapy vignette [F_(3,__17.62)_ = 7.73, *p* = .001]: ChatGPT-3 had significantly higher confidence than ChatGPT-4 and Claude 2 (*p* < .01) but was not different from Bard (*p* = .290).

##### 3.2.3.2 Self-direction vs tradition

In both the End-of-Life Dialysis Care and Contraception vignettes, the LLMs overwhelmingly chose the self-direction answer (100 %), and this was significantly different than chance level (.5; binomial test; *p* < .001 for both vignettes). As there was no variation in answers, there was no need to test the differences between the LLMs.

We examined the association between the confidence scale of both answers (Fig. 5C and D). In the End-of-Life Dialysis Care vignette, we found a significant model for the association between the confidence scale of both answers, which explained 28.43 % of the variance (R^2^_Marginal_; LRT X^2^_(1)_ = 9.03, *p* = .002), and a significant negative coefficient (B = −.64, t_(12.87)_ = -3.22, *p* = .006). In the Contraception vignette, we found a significant model, which explained 62.30 % of the variance (R^2^_Marginal_; LRT X^2^_(1)_ = 50.95, *p* < .001), and a significant negative coefficient (B = −.88, t_(35.69)_ = −10.79, *p* < .001). These results suggest that the higher the confidence in the self-direction answer, the lower the confidence in the tradition answer.

We then examined the differences in the confidence in the chosen answer between the LLMs, in each of the four values and in both vignettes (Fig. 6C and D). The LLMs differed in the self-direction answer in the End-of-Life Dialysis Care vignette [F_(3,__36)_ = 24.93, *p* < .001]: Claude 2 and Bard had significantly lower and higher confidence, respectively, than all the other LLMs (all comparisons, except for the ChatGPT comparisons, were significant at significant level of at least *p* < .05). The LLMs also differed in the self-direction answer in the Contraception vignette [F_(3,__19.19)_ = 6.04, *p* = .004]: Bard had significantly lower confidence than both ChatGPT-4 (*p* = .022) and ChatGPT-3.5 (*p* = .002) but was not different from Claude 2 (*p* = .367).

The LLMs differed in the tradition answer in the End-of-Life Dialysis Care vignette [F_(3,__36)_ = 6.68, *p* = .001]: Bard had significantly lower confidence than ChatGPT-4 (*p* = .003), ChatGPT-3.5 (*p* = .04) and Claude 2 (*p* = .001). However, no difference was found in the Contraception vignette [F_(3,__17.72)_ = 2.61, *p* = .083].

## 4 Discussion

This study aimed to elucidate the implicit value-like constructs embedded within four leading LLMs - Claude, Bard, GPT-3.5, and GPT-4 - using Schwartz's theory of basic human values as an analytical framework. Specifically, we sought to delineate whether each model exhibits a distinct value-like profile, assess alignment with general population values across numerous cultures, and evaluate the impact of latent value-like constructs on clinical decision-making dilemmas in primary care.

Before presenting our findings and their potential implications, it is crucial to address several important methodological and theoretical considerations that underpin our study and influence the interpretation of our results. It is important to note that the application of Schwartz's theory of basic values to analyze LLMs raises several methodological and theoretical issues [14]. There is an inherent anthropomorphic assumption in applying a human value model to artificial systems. While this approach enables us to compare LLM functions to human behavior, it may also lead to misinterpretations of AI capabilities.

Despite these conceptual distinctions, analyzing these value-like constructs provides valuable insights into the operational tendencies of LLMs. By comparing these constructs to human value systems, we can better understand the potential implications of deploying these models in diverse cultural contexts.

Our findings demonstrate that each LLM has a unique value-like profile. A comparison to the median rankings extracted from a heterogeneous international sample highlighted that universalism and self-direction were attributed greater significance by most models while power and tradition were attributed less significance.

We also provided preliminary evidence that the LLMs' recommend clinical solutions, when confronted with prototypical professional quandaries, affect its embedded value-like profile. This suggests that the value-like profile generated during the alignment process impacts the choice selection and confidence of the output. Further inspection revealed that GPT-3.5 occasionally deviated from the predominant decision-making pattern displayed by the other models. Overall, these findings have potential implications for the integration of LLMs within healthcare settings, particularly primary care, necessitating meticulous elucidation of values-based tensions, biases, and influences on guidance tailored to practitioners.

The finding that every model had a unique value-like profile suggests variability in implicit values. This variability can lead to different generated output, according to the chosen LLM, and must therefore be taken into account when choosing an LLM for application in healthcare environment [38]. The finding warrants careful consideration due to LLMs potential direct influences on guidance for clinicians and assistance to patients [[10], [11], [12]].

Moreover, the findings raise profound questions regarding potential Western-centric perspectives and biases embedded within the computational architectures of LLMs [6,[45], [46], [47]]. The prioritization of values linked to universalism and self-direction over power and tradition aligns with prevalent cultural dynamics across Western societies [48]. Hence, the observed value "preferences" may constitute indicative markers of implicit cultural assumptions permeating the development and training procedures of LLMs [14].

These current findings strengthen the notion that LLMs integration in primary care may have implications and necessitates a meticulous inspection of the concordance between embedded value-like hierarchies and the cultural outlooks of intended diverse users. Specifically, LLMs promoting values of individualism and egalitarianism may receive heightened trust and compliance among Western physicians and patients, whereas models favoring conventionalism and hierarchies might demonstrate a better fit for traditional communities [[49], [50], [51], [52]].

### 4.1 Potential applications and implications for primary care

The findings of this study underscore the necessity for cautious integration of LLMs in primary care settings. The Western bias identified in these models has the potential to significantly influence treatment recommendations and patient interactions, particularly when dealing with individuals from diverse cultural backgrounds [53].

In the context of primary care, the implications of these biases are multifaceted. First, there is a pressing need to develop mechanisms for culturally adapting LLM recommendations. This adaptation should not compromise medical standards but rather enhance them by incorporating cultural sensitivity [14,54]. For example, an LLM's advice on lifestyle changes or treatment adherence strategies might need to be tailored to align with the patient's cultural norms and values, thereby increasing the likelihood of compliance and positive health outcomes.

Moreover, the training of primary care physicians in the use of LLMs must evolve to include skills in identifying and mitigating potential biases. Physicians should be equipped to critically evaluate LLM outputs, recognizing when these might reflect Western-centric values that may not be universally applicable. This critical approach will enable physicians to use LLMs as supportive tools while maintaining their clinical judgment and cultural awareness [55,56].

The development of culturally tailored versions of LLMs for use in primary care represents another potential avenue for addressing the identified biases. These specialized models could be trained on diverse cultural datasets, incorporating medical knowledge and practices from various global healthcare systems. Such an approach could yield LLMs that are more adept at providing culturally appropriate recommendations across a spectrum of patient populations [57].

Furthermore, these findings call for extended research to examine the real-world impact of these biases on treatment outcomes in primary care settings. Longitudinal studies comparing patient outcomes when physicians use culturally biased versus culturally adapted LLMs could provide valuable insights into the practical implications of these biases [55].

Lastly, the issue of transparency with patients regarding the use of LLMs in their care is paramount. Developing mechanisms to inform patients about the involvement of AI in their treatment planning, including potential biases, is crucial for maintaining trust and enabling informed decision-making. This transparency could take the form of clear communication protocols or patient education materials that explain the role and limitations of LLMs in the care process [58].

In conclusion, the judicious implementation of LLMs in primary care, informed by the findings of this study, has the potential to enhance the quality of care while ensuring cultural sensitivity and ethical practice. However, this integration must be approached with caution, continuous evaluation, and a commitment to addressing the Western-centric biases identified in this research. By doing so, the medical community can harness the power of these advanced technologies while mitigating potential negative impacts on diverse patient populations.

### 4.2 Addressing western-centric bias in LLMs

The Western-centric bias identified in LLMs presents a significant challenge for their application in diverse healthcare settings. While technological solutions have been proposed to mitigate this bias, it is crucial to consider both the development of these models and their practical implementation in healthcare contexts.

At the model development level, several approaches have been suggested to reduce Western-centric bias. These include the use of de-biasing algorithms designed to adjust training data to better represent non-Western perspectives and the implementation of adversarial training methods to challenge the model with counterexamples that expose cultural biases [59]. Frameworks such as Fairness Flow [60] have also been proposed to systematically detect and mitigate bias in machine learning models. While these technical solutions show promise, their effectiveness in eliminating deep-seated cultural biases remains to be fully evaluated in the context of healthcare applications [61].

An alternative approach acknowledges that Western-centric bias may be inherent to these models due to their training data and development context. This perspective suggests that instead of attempting to eliminate bias entirely, efforts should focus on understanding the nature and extent of this bias and developing guidelines for appropriate use of LLMs in healthcare settings. This approach emphasizes the importance of cultural competence among healthcare providers, enabling them to critically evaluate and contextualize LLM outputs within diverse cultural frameworks [62].

Ultimately, addressing Western-centric bias in LLMs requires a multifaceted approach. This includes ongoing efforts to improve model development and training processes as well as educating healthcare providers about these tools' limitations and potential biases. Future research should focus on evaluating the effectiveness of various de-biasing techniques in healthcare contexts and developing best practices for the ethical and culturally sensitive implementation of LLMs in diverse healthcare settings.

### 4.3 Ethical considerations and practical steps for culturally aware LLM implementation

The integration of LLMs in diverse cultural contexts within healthcare settings poses significant ethical challenges that demand careful consideration. Central to these challenges is the need to balance the potential benefits of AI-assisted healthcare with respect for cultural diversity and individual autonomy.

Ethical principles guiding the cultural adaptation of LLMs should include respect for cultural diversity, transparency in decision-making processes, non-discrimination, beneficence, and justice. Respect for cultural diversity requires LLMs to recognize and accommodate various cultural perspectives on health and healthcare. Transparency involves clear communication about the use of AI in healthcare decisions and its potential biases. Non-discrimination ensures that the adaptation process does not perpetuate or exacerbate existing healthcare disparities. Beneficence and justice principles should guide the process of improving health outcomes equitably across all cultural groups [63,64].

Implementing these ethical principles in clinical settings requires a structured approach. We thus propose the following framework for the ethical deployment of culturally aware LLMs in healthcare.

1. Cultural competence training: Healthcare providers should receive training on the use of LLMs, including their limitations and potential biases, to ensure appropriate interpretation and application of AI-generated recommendations.
2. Ethical review process: An ethics review board should be established to oversee the development and deployment of LLMs in healthcare settings, ensuring adherence to ethical guidelines and cultural sensitivity.
3. Continuous monitoring and evaluation: Systems should be implemented for the ongoing assessment of LLM performance across different cultural contexts, with mechanisms for rapid correction of identified biases or errors.
4. Patient engagement: Protocols should be developed for informing patients about the use of AI in their care, obtaining informed consent, and providing channels for feedback and concerns.
5. Adaptive guidelines: Flexible ethical guidelines that can evolve with advancements in LLM technology and changing societal norms should be created.

This framework aims to ensure that the implementation of LLMs in healthcare is not only technologically advanced but also ethically sound and culturally sensitive. By addressing these ethical considerations and providing practical steps for implementation, we can work toward harnessing the potential of LLMs to improve healthcare outcomes while respecting the diverse cultural values of patients and communities.

### 4.4 Limitations and future directions

While our study provides valuable insights into the value-like constructs embedded in LLMs, it is important to acknowledge several limitations. Our analysis was confined to four commercial LLMs, which may not fully represent the diverse landscape of AI capabilities and potential biases. The exclusion of open-source models and models developed in non-Western contexts limits the generalizability of our findings and highlights the need for more comprehensive studies in the future.

Additional limitation in our approach lies in the application of human-centric value theories to AI systems. This anthropomorphic assumption may lead to misinterpretations of LLM capabilities and decision-making processes, necessitating caution in drawing parallels between human values and AI-generated outputs.

The rapidly evolving nature of AI technologies presents another significant limitation. Our results represent a snapshot in time, and the capabilities and biases of LLMs may change substantially over short periods. This dynamic landscape could affect the stability and longevity of our conclusions, particularly in healthcare settings where AI integration is advancing quickly. The cross-sectional nature of our study does not capture potential changes in LLM value profiles over time or in response to different types of input data, which may impact the long-term reliability of our findings.

To address these limitations and advance our understanding of LLMs in healthcare contexts, future research should pursue several key directions. Longitudinal studies tracking changes in LLM value profiles over time and across different versions or training iterations could provide valuable insights into the stability of value-like constructs in these systems. Expanding the range of LLMs studied, including open-source models and models developed in non-Western contexts, could offer a more comprehensive understanding of value-like constructs across diverse AI systems.

Future work should also explore alternative theoretical frameworks that may better capture the unique aspects of artificial agents and reduce reliance on anthropomorphic assumptions. Investigating the practical impact of LLM value profiles on patient outcomes and healthcare provider decision-making in real-world clinical settings could bridge the gap between theoretical findings and practical applications.

Examining how LLMs' value-like constructs and decision-making processes change in response to different types of input data or prompts could provide insights into the flexibility and adaptability of these systems in varied healthcare scenarios. Implementing ongoing assessment strategies for LLMs in healthcare applications, including regular re-evaluation of their value profiles and decision-making patterns, could ensure continued alignment with ethical standards and cultural sensitivities.

Finally, research into methods for integrating ethical considerations directly into the architecture and training processes of LLMs, rather than relying solely on post-hoc alignment techniques, could lead to the development of more inherently ethical AI systems for healthcare.

By addressing these limitations and pursuing these research directions, future studies can build upon our findings to develop more robust, culturally sensitive, and ethically aligned LLMs for use in healthcare settings. This ongoing research is crucial for ensuring that as AI technologies continue to advance, their integration into healthcare systems remains both beneficial and ethically sound.

### 4.5 Conclusion

This study's findings highlight significant implications regarding the integration of LLMs in primary care. Foremost, each model was shown to exhibit a relatively distinct internal value-like profile shaping its recommendations. This mandates careful deliberation when selecting aids to uphold organizational values and avoid alienating communities. Specifically, the prioritization of dimensions like universalism and self-direction risks taking on Western assumptions. However, variations between models on dimensions like power and conformity suggest that some may be better suited for traditional cultures. Meticulous alignment processes are thus imperative prior to deployment in care settings that serve diverse populations.

Moreover, the presence of inconsistencies in decision-making, particularly evident in GPT-3.5, suggests that further robust vetting is needed before LLMs can be relied on as judgment aids. While the models show promise, achieving equitable translation still demands progress on the elucidation of biases.

Thus, beyond demonstrating sheer predictive accuracy, the implementation of LLMs as clinical decision supports requires the confirmation of context-appropriate guidance. We propose cultivating adaptable systems with configurable parameters that align outputs with nuanced communal needs and norms. The prioritization of cultural customization promises to accelerate the inclusive and ethical adoption of LLMs in primary care, which will benefit patients the world over [[65], [66], [67]].

As AI technologies continue to advance, it is crucial that we maintain a critical and reflective approach to their integration in healthcare. The ethical implications of embedding value-like constructs in LLMs extend beyond technical considerations, touching on fundamental questions of cultural respect, patient autonomy, and global health equity. By addressing these challenges head-on, we can work toward a future in which AI augments and enhances healthcare delivery in a manner that is both ethically sound and culturally sensitive.

## Patient and public involvement

No patient involved.

## Availability of data and materials

The research data is available from the authors upon request.

## CRediT authorship contribution statement

**Dorit Hadar-Shoval:** Conceptualization, Formal analysis, Investigation, Methodology, Project administration, Supervision, Writing – original draft, Writing – review & editing. **Kfir Asraf:** Conceptualization, Data curation, Formal analysis, Investigation, Methodology, Validation, Visualization, Writing – original draft, Writing – review & editing. **Shiri Shinan-Altman:** Conceptualization, Investigation, Methodology, Writing – original draft, Writing – review & editing. **Zohar Elyoseph:** Conceptualization, Data curation, Formal analysis, Methodology, Project administration, Writing – original draft, Writing – review & editing. **Inbar Levkovich:** Conceptualization, Data curation, Methodology, Project administration, Writing – original draft, Writing – review & editing.

## Declaration of generative AI and AI-assisted technologies in the writing process

During the preparation of this work, the authors used Claude.AI (by Anthropic), and ChatGPT 4 (by OpenAI) to refine the fluency of the English language and accuracy of the academic writing. After using these tools, the authors reviewed and edited the content as needed and took full responsibility for the content of the publication.

## Declaration of competing interest

The authors declared no potential conflicts of interest with respect to the research, authorship, and/or publication of this article.

## References

[1] Elyoseph Z., Hadar-Shoval D., Asraf K., Lvovsky M. (2023). ChatGPT outperforms humans in emotional awareness evaluations. Front. Pyschol..

[2] Elyoseph Z., Levkovich I. (2023). Beyond human expertise: the promise and limitations of ChatGPT in suicide risk assessment. Front. Pyschol..

[3] Elyoseph Z., Levkovich I., Shinan-Altman S. (2024). Assessing prognosis in depression: comparing perspectives of AI models, mental health professionals and the general public. Fam. Med. Community Health.

[4] Elyoseph Z., Refoua E., Asraf K., Lvovsky M., Shimoni Y., Hadar-Shoval D. (2023). Capacity of generative artificial intelligence to interpret human emotions from visual and textual data: pilot evaluation study. JMIR Ment. Health.

[5] Levkovich I., Elyoseph Z. (2023). Identifying depression and its determinants upon initiating treatment: ChatGPT versus primary care physicians. Fam. Med. Community Health.

[6] Hadar-Shoval D., Elyoseph Z., Lvovsky M. (2023). The plasticity of ChatGPT's mentalizing abilities: personalization for personality structures. Front. Psychiatr..

[7] Albahri A.S., Duhaim A.M., Fadhel M.A., Alnoor A., Baqer N.S., Alzubaidi L., Albahri O.S., Alamoodi A.H., Bai J., Salhi A. (2023). A systematic review of trustworthy and explainable artificial intelligence in healthcare: assessment of quality, bias risk, and data fusion. Inf. Fusion.

[8] Kumar P., Chauhan S., Awasthi L.K. (2023). Artificial intelligence in healthcare: review, ethics, trust challenges & future research directions. Eng. Appl. Artif. Intell..

[9] Terra M., Baklola M., Ali S., El-Bastawisy K. (2023). Opportunities, applications, challenges and ethical implications of artificial intelligence in psychiatry: a narrative review, Egypt. J. Neurol. Psychiat. Neurosurg..

[10] Haug C.J., Drazen J.M. (2023). Artificial intelligence and machine learning in clinical medicine. N. Engl. J. Med..

[11] Kooli C., Al Muftah H. (2022). Artificial intelligence in healthcare: a comprehensive review of its ethical concerns. Technol. Sustain.

[12] McCradden M., Hui K., Buchman D.Z. (2023). Evidence, ethics and the promise of artificial intelligence in psychiatry. J. Med. Ethics.

[13] Schwartz S.H., Cieciuch J. (2022). Measuring the refined theory of individual values in 49 cultural groups: psychometrics of the revised portrait value questionnaire. Assessment.

[14] Hadar-Shoval D., Asraf K., Mizrachi Y., Haber Y., Elyoseph Z. (2024). Assessing the alignment of large language models with human values for mental health integration: cross-sectional study using Schwartz's theory of basic values. JMIR Mental Health.

[15] Schwartz S.H. (1992). Universals in the content and structure of values: theoretical advances and empirical tests in 20 countries. Adv. Exp. Soc. Psychol..

[16] Moyo M., Shulruf B., Weller J., Goodyear-Smith F. (2019). Effect of medical students' values on their clinical decision-making. J. Prim. Health Care.

[17] Schwartz S.H., Bardi A. (2001). Value hierarchies across cultures: taking a similarities perspective, J. Cross-Cult. Psychol..

[18] Schwartz S.H., Cieciuch J., Vecchione M., Torres C., Dirilen-Gumus O., Butenko T. (2017). Value tradeoffs propel and inhibit behavior: validating the 19 refined values in four countries. Eur. J. Soc. Psychol..

[19] Schwartz S.H. (1994). Are there universal aspects in the structure and contents of human values?. J. Soc. Issues.

[20] Schwartz S.H., Cieciuch J., Vecchione M., Fischer R., Ramos A., Konty M. (2012). Refining the theory of basic individual values. J. Pers. Soc. Psychol..

[21] Kaya A., Boz I. (2019). The development of the professional values model in nursing. Nurs. Ethics.

[22] Rose T., Nies M.A., Reid J. (2018). The internalization of professional nursing values in baccalaureate nursing students. J. Prof. Nurs..

[23] Kantek F., Kaya A. (2017). Professional values, job satisfaction, and intent to leave among nursing managers. J. Nurs. Res..

[24] Ravari A., Bazargan-Hejazi S., Ebadi A., Mirzaei T., Oshvandi K. (2013). Work values and job satisfaction: a qualitative study of Iranian nurses. Nurs. Ethics.

[25] Jasemi M., Cheraghi R., Azimzadeh R., Namadi F. (2020). The relationship between personality characteristics and adherence to professional values among nursing students. Nurs. Midwifery Stud..

[26] Ogunyemi A.O. (2020). Life satisfaction and personal values as mediators of work engagement and turnover intention among medical officers in south-west, Nigeria. KIU J. Soc. Sci..

[27] Skrzypek M., Turska D., Marzec A., Szczygieł K. (2020). Personality traits and personal values as retail pharmacy choice predictors in the context of pharmaceutical care requirements. Res. Soc. Adm. Pharm..

[28] Merriman C., Chalmers L., Ewens A., Fulford B., Gray R., Handa A., Westcott L. (2020). Values-based interprofessional education: how interprofessional education and values-based practice interrelate and are vehicles for the benefit of patients and health and social care professionals. J. Interprof. Care.

[29] Pomare C., Long J.C., Churruca K., Ellis L.A., Braithwaite J. (2020). Interprofessional collaboration in hospitals: a critical, broad-based review of the literature. J. Interprof. Care.

[30] Ashcroft R.E., Dawson A., Draper H., McMillan J. (2007).

[31] Schwartz S.H. (2012). An overview of the Schwartz theory of basic values. Online Readings in Psychology and Culture.

[32] Kørup A.K., Søndergaard J., Lucchetti G., Ramakrishnan P., Baumann K., Lee E., Frick E., Büssing A., Alyousefi N.A., Karimah A., Schouten E., Wermuth I., Hvidt N.C. (2019). Religious values of physicians affect their clinical practice: a meta-analysis of individual participant data from 7 countries. Medicine.

[33] Cheraghi-Sohi S., Calnan M. (2013). Discretion or discretions? Delineating professional discretion: the case of English medical practice. Soc. Sci. Med..

[34] Hackett J., Glidewell L., West R., Carder P., Doran T., Foy R. (2014). “Just another incentive scheme”: a qualitative interview study of a local pay-for-performance scheme for primary care. BMC Fam. Pract..

[35] Lester H., Matharu T., Mohammed M.A., Lester D., Foskett-Tharby R. (2013). Implementation of pay for performance in primary care: a qualitative study 8 years after introduction. Br. J. Gen. Pract..

[36] Wyatt T.R., Rockich-Winston N., White D., Taylor T.R. (2021). “Changing the narrative”: a study on professional identity formation among Black/African American physicians in the US. Adv. Health Sci. Educ..

[37] Abu-Ras W., Senzai F., Laird L., Decker E. (2022). The influence of religious identity, culture, and values on the practice of American Muslim physicians. Soc. Sci..

[38] Hordern J. (2016). Religion and culture. Medicine.

[39] Stahl J.E., Nelson W.A. (2022). Applying the peter parker principle to healthcare. Camb. Q. Healthc. Ethic..

[40] Alfahmi M.Z. (2022). Patients' preference approach to overcome the moral implications of family-centred decisions in Saudi medical settings. BMC Med. Ethics.

[41] Eves M.M., Danziger P.D., Farrell R.M., Cole C.M. (2015). Conflicting values: a case study in patient choice and caregiver perspectives. Narrat. Inq. Bioeth..

[42] Safdari M., Serapio-García G., Crepy C., Fitz S., Romero P., Sun L., Abdulhai M., Faust A., Mataric M. (2023). Personality traits in large language models. ArXiv Preprint.

[43] Caldwell A.R. (2022). SimplyAgree: an R package and jamovi module for simplifying agreement and reliability analyses. J. Open Source Softw..

[44] Shieh G. (2020). Assessing agreement between two methods of quantitative measurements: exact test procedure and sample size calculation. Stat. Biopharm. Res..

[45] Cao Y., Zhou L., Lee S., Cabello L., Chen M., Hershcovich D. (2023). Assessing cross-cultural alignment between ChatGPT and human societies: an empirical study.

[46] Johnson R.L., Pistilli G., Menédez-González N., Duran L.D.D., Panai E., Kalpokiene J., Bertulfo D.J. (2022). The ghost in the machine has an American accent: value conflict in GPT-3. ArXiv Preprint.

[47] Miotto M., Rossberg N., Kleinberg B. (2022). Proceedings of the Fifth Workshop on Natural Language Processing and Computational Social Science.

[48] Mattar S., Gellatly R. (2022). Refugee mental health: culturally relevant considerations. Curr. Opin. Psychol..

[49] Kirmayer L.J. (2019). The politics of diversity: pluralism, multiculturalism and mental health. Transcult. Psychiatry.

[50] Havaldar S., Rai S., Singhal B., Liu L., Guntuku S.C., Ungar L. (2023). Proceedings of the 13th Workshop on Computational Approaches to Subjectivity, Sentiment, & Social Media Analysis, Toronto, Canada.

[51] Liu Y., Yao Y., Ton J., Zhang X., Cheng R.G.H., Klochkov Y., Taufiq M.F., Li H. (2023). Trustworthy LLMs: a survey and guideline for evaluating large language models' alignment. ArXiv Preprint.

[52] Naous T., Ryan M.J., Xu W. (2023). Having beer after prayer? Measuring cultural bias in large language models. ArXiv Preprint.

[53] Kotek H., Sun D.Q., Xiu Z., Bowler M., Klein C. (2024). Protected group bias and stereotypes in large language models.

[54] Lv X., Zhang X., Li Y., Ding X., Lai H., Shi J. (2024). Leveraging large language models for improved patient access and self-management: assessor-blinded comparison between expert- and AI-generated content. J. Med. Internet Res..

[55] Kämmer J.E., Hautz W.E., Krummrey G., Sauter T.C., Penders D., Birrenbach T., Bienefeld N. (2024). Effects of interacting with a large language model compared with a human coach on the clinical diagnostic process and outcomes among fourth-year medical students: study protocol for a prospective, randomised experiment using patient vignettes. BMJ Open.

[56] Pressman S.M., Borna S., Gomez-Cabello C.A., Haider S.A., Haider C.R., Forte A.J. (2024). Clinical and surgical applications of large language models: a systematic review. J. Clin. Med..

[57] Sato K., Kaneko H., Fujimura M. (2024). Reducing cultural hallucination in non-English languages via prompt engineering for large language models. OSF Preprints.

[58] Park H.J. (2024). Patient perspectives on informed consent for medical AI: a web-based experiment, Digit. Health.

[59] Kim P.T. (2022). Race-aware algorithms: Fairness, nondiscrimination and affirmative action. Calif. Law Rev..

[60] Chakraborty J., Tu H., Majumder S., Menzies T. (May 21–29, 2022).

[61] Tokayev K.J. (2023). Ethical implications of large language models: a multidimensional exploration of societal, economic, and technical concerns. Int. J. Soc. Anal..

[62] Hadar-Shoval D., Haber Y., Tal A., Simon T., Elyoseph T., Elyoseph Z. (2024). Transforming perceptions: exploring the multifaceted potential of generative AI for people with cognitive disabilities. JMIR Preprints.

[63] Arefin S. (2024). AI revolutionizing healthcare: innovations, challenges, and ethical considerations, MZ J. Artif. Intell..

[64] Hasanah L.N. (2023). Leveraging AI to address language barriers in healthcare: ethical considerations and implementation strategies. JHASR.

[65] Garg R.K., Urs V.L., Agarwal A.A., Chaudhary S.K., Paliwal V., Kar S.K. (2023). Exploring the role of ChatGPT in patient care (diagnosis and treatment) and medical research: a systematic review. Health Promot. Perspect..

[66] Levkovich I., Elyoseph Z. (2023). Suicide risk assessments through the eyes of chatgpt-3.5 versus ChatGPT-4: vignette study, JMIR ment. Health.

[67] Moyo M., Goodyear-Smith F.A., Weller J., Robb G., Shulruf B. (2016). Healthcare practitioners' personal and professional values. Adv. Health Sci. Educ. Theory Pract..

